# Editorial: Life under pressure: microbial adaptation and survival in high pressure environments

**DOI:** 10.3389/fmicb.2026.1804037

**Published:** 2026-02-25

**Authors:** Mohamed Jebbar, Xue-Gong Li

**Affiliations:** 1 Univ Brest, Ifremer, CNRS, EMR 6002 BIOMEX, BEEP, Plouzané, France; 2 Laboratory of Deep-Sea Microbial Cell Biology, Institute of Deep-Sea Science and Engineering, Chinese Academy of Sciences, Sanya, China

**Keywords:** adaptation, high hydrostatic pressure, membrane adaptation, prokaryote, transcriptional regulation

A quantitative overview of Earth's biomass has long been lacking, despite its importance for understanding the biosphere. The first global estimate—approximately 550 gigatons of carbon (Gt C)—reveals stark disparities across life forms (Bar-On et al., 2018). Plants dominate at nearly 450 Gt C, mostly on land, while animals account for only about 2 Gt C, largely in the ocean. Bacteria (70 Gt C) and archaea (7 Gt C) reside predominantly in the deep subsurface.

This vast subsurface biosphere consists mainly of prokaryotes (Flemming and Wuertz, 2019), totaling roughly 1.2 × 10^30^ cells distributed across five major habitats: deep oceanic waters, upper oceanic sediments, deep continental subsurface, soils, and the open ocean. These environments are typically extreme, characterized by nutrient scarcity, wide temperature ranges, and high hydrostatic pressure (HHP).

HHP plays a critical role in these habitats. Around a hundred prokaryotes, isolated from various temperature regimes of the deep biosphere, have been found to be piezophilic—meaning they require HHP for optimal growth. Several major questions remain unanswered: What is the origin of piezophiles? What are the molecular mechanisms underlying their adaptation to pressure? The nine articles of this Research Topic expand current knowledge on cellular, molecular, and adaptive responses to HHP in piezosensitive and piezophilic prokaryotes.

Cui et al., investigated how HHP disrupts cell division in model microorganisms. Through analysis of the hadal piezophile *Shewanella benthica* DB21MT-2, five critical residues in the N-terminal GTPase domain of FtsZ were identified as essential for high-pressure adaptation, underscoring the functional importance of this domain. These findings advance our understanding of microbial cell division under extreme conditions.

Coffin et al., focus on the pressure-dependent heat shock response in *E. coli* strains using GFP promoter fusions and single-cell fluorescence microscopy. They quantified the transcriptional upregulation of *rpoH, rpoE, dnaK*, and *groEL* following pressure shock in both piezosensitive *E. coli* and the piezotolerant strain AN62. The results reveal distinct responses in the pressure-adapted strain, suggesting RpoE may act as a pressure sensor. These findings link pressure adaptation to RNA polymerase function and highlight stochasticity in stress responses.

Tamby et al., investigate how bacterial and archaeal ether-bonded lipids affect *E. coli* membrane robustness under high temperature and HHP. Only bacterial ether lipids enhance robustness, especially under combined stress. The results emphasize that temperature and HHP together critically affect survival, advancing understanding of membrane adaptation and the “lipid divide.” This suggests such abiotic factors may have influenced early marine evolution.

Moalic et al., showed in the piezophilic hyperthermophilic archaeon *Thermococcus barophilus* that the redox regulator SurR and HHP co-regulate energy metabolism genes. Pressure modulates gene expression, while SurR activates hydrogenogenic genes regardless of sulfur availability. Unlike non-piezophilic species, *T. barophilus* uses a sulfur-independent SurR mechanism for deep-sea adaptation. Also, Jiao et al., show that HHP enhances both sulfur reduction and growth in *Thermococcus aciditolerans* SY113 independently of SurR, highlighting the essential role of sulfur reduction to pressure adaptation. These findings advance our understanding of extremophile biology and provide insights for biotechnological and astrobiological applications under high-pressure conditions.

Qiu and Tang, examine how the deep-sea bacterium *Shewanella eurypsychrophilus* YLB-09 adapts metabolically to HHP. Through combined metabolomics and transcriptomics, they found that HHP markedly reshapes energy, amino acid, and glycerolipid metabolism. Key adaptations include a switch to TMAO respiration, altered amino acid profiles, and adjusted membrane fluidity and composition. These metabolic adjustments enable YLB-09 to thrive under extreme pressure, offering insights into microbial survival strategies and supporting the development of industrially useful enzymes from extremophiles.

Li et al., studied the piezotolerant fungus *Purpureocillium lilacinum* FDZ8Y1 from the Mariana Trench. Strain FDZ8Y1 withstands pressures up to 110 MPa and shows antibacterial, antitumor, and nematicidal activity. Genomic and transcriptomic analyses reveal its metabolic versatility in carbon, nitrogen, and sulfur cycling, along with the activation of fatty acid metabolism, antioxidant defenses, and secondary metabolite biosynthesis under HHP. These findings reveal the significant potential of hadal fungi for producing novel bioactive compounds.

Zhang et al., compared sediment microbial communities in the Diamantina and Kermadec trenches using high-throughput sequencing. They found that both trenches exhibit distance–decay patterns, stochastic community assembly processes, and complex interspecific interactions that likely maintain community stability. Functional redundancy was also observed across taxonomic groups. These findings highlight the important roles of stochastic processes and functional redundancy in hadal ecosystems, advancing our understanding of microbial community assembly in extreme environments.

Del Moral et al., focus on Europa's potentially habitable sub-ice ocean, using Earth-based analogs to assess habitability. They employed Canada's Basque Lake No. 2,—a cold, hypersaline, Mg-Na-SO4-rich environment—as an analog for Europa's ice-ocean interface. Lake microorganisms were cultured in Europa-like fluid under progressively higher pressures (0.2–30 MPa), leading to the isolation of *Pseudodesulfovibrio* sp. OU_01 at 30 MPa. These findings advance our understanding of potential habitability and biosignature formation on icy moons.

In conclusion, the deep biosphere is marked by extreme pressure, temperature, and nutrient scarcity, hosting most of Earth's bacteria and archaea, revealing a hidden biosphere adapted to challenging conditions (Jebbar et al., 2020). Recent research has uncovered how microbes thrive under high hydrostatic pressure (HHP) (Ando et al., 2021; Peters et al., 2025). The studies presented in this Research Topic reveal that Piezophilic species, which require HHP for growth, have evolved distinctive molecular adaptations. Notably, specific residues in essential cell division proteins like FtsZ enhance pressure tolerance, while heat shock responses and transcriptional regulators (e.g., RpoE) help manage stress. Membrane composition also matters, with certain lipids improving robustness under combined HHP and high temperature. In deep-sea archaea, pressure and redox regulators co-control energy metabolism, particularly sulfur reduction, which is vital for survival in these environments. Metabolomic studies further show that piezophiles adjust their energy pathways, respiration, and membrane fluidity for adaptation. Deep-sea trenches, such as the Diamantina and Kermadec, host microbial communities with lower biodiversity but high functional redundancy, maintained through complex interactions. These findings not only advance our understanding of life in extreme environments but also inform astrobiology, as microbes from Earth's analog sites can grow at pressures relevant to Europa's ocean. This research underscores life's resilience and opens new possibilities for biotechnology and the search for extraterrestrial life.
